# Valvular Lesions without Bruit

**Published:** 1909-03-06

**Authors:** 


### Medicine.

### VALVULAR LESIONS WITHOUT BRUIT.

It is fortunately true that in nearly every case in which the stethoscope reveals normal heart sounds the cardiac valves are healthy; and that in most cases of valvular heart disease there is a bruit or other stethoscopic evidence of the lesion.

Nevertheless it is quite important to remember that there are exceptions to these rules, and that serious valvular mischief is not necessarily excluded because there is no cardiac bruit. It is particularly in cases of *fungating endocarditis* that this is so; large masses of vegetations are sometimes found unexpectedly in the post-mortem room in a subject whose illness may have seemed very obscure, but whose cardiac sounds have always been perfectly normal. But even *fibrotic valvular disease* with actual organic stenosis may exist without bruit. One is familiar with this in certain cases of mitral stenosis, both when failure of compensation is considerable, and more rarely when there are no symptoms of cardiac mischief at all. It is not only in mitral stenosis, however, that bruits may be absent.

### A Case of Congenital Pulmonary Stenosis.

The following is an instance of *congenital heart disease*—pulmonary stenosis and perforated septum ventriculorum—in which there was no bruit. The congenital cyanosis at once led to a diagnosis of heart malformation; but as there was no bruit, one might have been led into making the definite assertion that there was no pulmonary stenosis had the autopsy not proved that there was. Cases of congenital heart disease with cyanosis but no bruit are familiar enough, and one is usually content to make the general diagnosis without specifying the precise lesion. It illustrates our present point, however, when one finds valvular stenosis present in some of these cases in spite of the absence of all bruit: although in the great majority of cases of cardiac malformation with stenosis of the pulmonary valve there is a loud rumbling systolic bruit, heard best in the second or third left space close to the stenum, with a thrill in the same locality.

The child in question was fifteen months old when it came under observation. It had been difficult to rear, was undersized, with clubbed fingers, clubbed toes, and a colour typical of morbus cœruleus. The cyanosis and lividity increased when the child made any effort, such as crying, the lips and finger nails going a deep violet hue. As in all such cases there was a marked hyperglobulæmia, the red corpuscles numbering 7,520,000 per c.mm., and the white corpuscles 14,800.

The only cardiac abnormality to be made out in the ordinary examination of the thorax was the rapid rate of the heart-beat; there was neither thrill nor bruit. The cause of the parents seeking medical advice was diarrhœa, which had led to rapid wasting. There was pyrexia, and a day or two later a cough set in, with the physical signs of bronchitis in both lungs. Most unfortunately, when all seemed to be going well notwithstanding the bronchitis, measles developed, broncho-pneumonia rapidly supervened, and the infant died. At the post-mortem examination the lungs were found studded with areas of lobular pneumonia and collapse. The remaining organs were healthy, except the heart.

### The Valvular Lesions Described.

The malformations which were found to be present in the heart and great vessels may be described under the headings of the pulmonary artery, the septum ventriculorum, and the aorta: for all the rest of the cardiac apparatus was, approximately, healthy.

The conus arteriosus of the right ventricle passed directly on into the pulmonary artery, but the latter at its origin was extremely narrow, measuring only 6 mm. in diameter, whereas the aorta at the corresponding level measured 13 mm. There were only two flaps to the pulmonary semilunar valve instead of three; unlike the condition met with in most cases of the kind, these were not united into a cone, but in themselves seemed not absolutely malformed; the deformity was in the wall of the artery—a narrowing of its entire lumen—rather than in the shape of the valve flaps. Beyond the valve the artery rapidly expanded to its normal size, divided into two in the normal way, and was distributed naturally to the lungs. It was doubtless partly because the stenosis was of the entire artery wall, rather than of the valve within the artery, that there was no bruit. And yet if stenosis is the cause of bruits there should have been one in this instance; for there was no doubt at all that here was present very marked stenosis.

The septum ventriculorum was almost complete, and in its lower part it was very thick and strong. In the typical spot, however, namely, in the pars membranacea, just below the aortic semilunar valve, there was a hole through it large enough to permit a big probe to pass. Even this had caused no bruit, so that the pressures in right and left ventricles cannot have been very unequal during life, one would suppose, or else blood would have been driven from one ventricle to the other through this hole during systole, and a bruit have resulted.

The aorta was itself of normal size, its valve was natural, and the ductus arteriosus was closed; but arising from the arch there were four branches instead of three—the first very large, the second less large, the third quite small, and the fourth intermediate in size between the third and second.

The case exemplifies the fact that fibrotic lesions which one would think ought to give rise to bruits do not necessarily do so, and that, therefore, absence of bruit is not synonymous with absence of lesion. The following is another instance in point, the lesion being fungating endocarditis, which sometimes causes extreme mischief and yet no bruit.

A Case of Fungating Endocarditis.

The patient was forty-three years old when, without any definite reason, he began to ail, and within a few days had to keep to his bed on account of lethargy. When seen on December 3 he was lying on his back, indifferent to all his surroundings, and answering questions only after they had been repeated several times. The man's habits were good; there had been neither alcoholism nor syphilis; there were none of the usual symptoms of cerebral tumour, and there seemed to be no cause for cerebral abscess. There was no paralysis, although from asthenia the legs gave way when the patient tried to get up and walk. The lungs were emphysematous, and there were non-consonating râles and rhonchi scattered generally over them and suggesting bronchitis. The heart presented no abnormal signs beyond the fact that the normal sounds were rather faint at the impulse; at the base they were quite clear and free from bruit. There was no canter rhythm nor any reduplication of any sound. There was no œdema of the ankles nor evidence of chronic heart disease. The liver was normal; there was no ascites; the urine contained neither sugar nor albumen. The temperature on admission was over 100° F. Next day the patient developed distinct aphasia, paralysis of the right arm, and paresis of the right leg. The optic discs were natural. On December 5 the symptoms remained about the same, pyrexia persisting, and the physical signs in heart and lungs remaining unchanged, although the pulse rate had risen to 120. Next morning, December 6, complete coma supervened and the patient died.

There were several points of interest at the post-mortem examination, but that which bore directly upon the subject just now under discussion was the condition of the mitral valve. The latter admitted three fingers to the first joint, and there was some thickening and induration of the edge as though from former endocarditis. In addition to this there was a large round pedunculated mass of fibrin the size of a walnut attached to its aortic cusp, the fungating lump being adherent to an older patch of endocarditis. The cause of the aphasia, hemiplegia, coma, and death had been cerebral embolism secondary to this extensive fungating endocarditis. The fact that so large a mass of fibrin could be attached to the mitral valve without giving rise to any stethoscopic evidence of its presence is a very good illustration of the fact that the absence of cardiac bruit is by no means synonymous with absence of valvular lesion.

